# The gut microbial metabolite trimethylamine N-oxide and cardiovascular diseases

**DOI:** 10.3389/fendo.2023.1085041

**Published:** 2023-02-07

**Authors:** Jing Zhen, Zhou Zhou, Meng He, Hai-Xiang Han, En-Hui Lv, Peng-Bo Wen, Xin Liu, Yan-Ting Wang, Xun-Chao Cai, Jia-Qi Tian, Meng-Ying Zhang, Lei Xiao, Xing-Xing Kang

**Affiliations:** ^1^ Department of Bioinformatics, School of Medical Informatics, Xuzhou Medical University, Xuzhou, Jiangsu, China; ^2^ School of Chemical Engineering and Technology, China University of Mining and Technology, Xuzhou, Jiangsu, China; ^3^ Department of Biochemical Pharmacy, School of Pharmacy, Naval Medical University, Shanghai, China; ^4^ Department of Gastroenterology and Hepatology, Shenzhen University General Hospital, Shenzhen University, Shenzhen, Guangdong, China

**Keywords:** gut microbiota, metabolite, cardiovascular disease, trimethylamine N-oxide, targeted therapy

## Abstract

Morbidity and mortality of cardiovascular diseases (CVDs) are exceedingly high worldwide. Researchers have found that the occurrence and development of CVDs are closely related to intestinal microecology. Imbalances in intestinal microecology caused by changes in the composition of the intestinal microbiota will eventually alter intestinal metabolites, thus transforming the host physiological state from healthy mode to pathological mode. Trimethylamine N-oxide (TMAO) is produced from the metabolism of dietary choline and L-carnitine by intestinal microbiota, and many studies have shown that this important product inhibits cholesterol metabolism, induces platelet aggregation and thrombosis, and promotes atherosclerosis. TMAO is directly or indirectly involved in the pathogenesis of CVDs and is an important risk factor affecting the occurrence and even prognosis of CVDs. This review presents the biological and chemical characteristics of TMAO, and the process of TMAO produced by gut microbiota. In particular, the review focuses on summarizing how the increase of gut microbial metabolite TMAO affects CVDs including atherosclerosis, heart failure, hypertension, arrhythmia, coronary artery disease, and other CVD-related diseases. Understanding the mechanism of how increases in TMAO promotes CVDs will potentially facilitate the identification and development of targeted therapy for CVDs.

## Introduction

The mammalian gut microbiota is the most abundant highly and diverse microbial community that resides in the gastrointestinal tract. The gut microbiome, as the “second genome”, plays an important role in dynamically integrating signals from the host and its environment, impacting both physiological homeostasis and disease emergence (1). Consequently, research on gut microbiota has become the focus of attention for many researchers. Meanwhile, in 2007, the National Institutes of Health (NIH) launched the Human Microbiome Project (HMP), aiming to elucidate microorganisms relevant to human health and disease (2). Cardiovascular diseases (CVDs) are associated with high morbidity and mortality, as well as considerable and increasing health-care costs. Based on the Global Burden of Disease (GBD) Study 2019, the total number of cases of CVDs almost doubled from 271 million in 1990 to 523 million in 2019; and the number of deaths from CVDs increased steadily from 12.1 million in 1990 to 18.6 million in 2019, accounting for 33% of all deaths globally (3). According to the European Society of Hypertension/Cardiology Guidelines, the occurrence and development of CVDs are combined effects of the risk factors, which include non-modifiable factors (age, gender, heredity) and modifiable factors (smoking, hypercholesterolemia, diabetes, lack of physical exercise, glucose intolerance, obesity), and target organ damage. Among the modifiable factors, smoking is the most important cardiovascular risk factor, and smoking cessation may be the most effective method for the prevention of CVDs among the general population (4). In recent years, metagenomic sequencing has verified that the gut microbiota is linked to many diseases, including CVDs (5).

Changes in gut microbiota alter the production of gut metabolites and thus affect the development of CVDs (6). Intestinal bacteria produce various bioactive metabolites, which can be absorbed into the enterohepatic circulation and enter the host blood circulation system, affecting host physiology directly or indirectly (7). Currently, there is growing evidence that some metabolites, such as short-chain fatty acids, bile acids (BAs), and trimethylamine N-oxide (TMAO), derived from gut microbiota, play important roles in the onset and progression of CVDs. Among them, TMAO has attracted attention recently, with evidence supporting an association between circulating levels of TMAO and increased risks of CVDs and higher all-cause mortality (8, 9). Hazen et al. reported the small-molecule metabolic profile of plasma through metabolomics and showed that the level of TMAO could be used to predict the risk of CVDs (10).

Although the role of TMAO in CVDs is well established, detailed understanding of how TMAO regulates the development and progression of CVDs has yet to be elucidated. Choline and L-carnitine are metabolized to disease-associated metabolite trimethylamine (TMA) by the gut microbiota. Subsequently, TMA is transported *via* the portal circulation to the liver where flavin monooxygenases (FMOs) catalyze the conversion of TMA into TMAO; however, the mechanism by which these precursors raise serum levels of TMAO and how changes in their levels contribute to CVDs remain unknown. This article reviews the involvement of TMAO in CVDs, which is helpful to understand the mechanism of TMAO leading to the development and progression of atherosclerosis, heart failure (HF), hypertension, and other CVDs. Furthermore, the solutions to regulate excessive TMAO levels from the perspective of diet, TMAO/TMA circulation, and gut microbiota regulation are reviewed, providing a promising targeted therapy for CVDs.

## The gut microbiota and CVDs

The human gut is colonized by approximately 100 trillion microorganisms–including beneficial bacteria, neutral bacteria, and pathogenic bacteria–which cooperate to maintain homeostasis of the gut microecology (11). Continuous improvements in next-generation sequencing technology and omic analysis methods, coupled with decreasing sequencing costs, have resulted in microbial genome sequencing becoming a rapid and convenient way to obtain detailed information on the relationship between structural changes of microbiota and human health based on gut microbiome data (7, 12). In the gut, *Bacteroidetes* and *Firmicutes* account for approximately 90% of the total gut microbial abundance, followed by *Proteobacteria* (5-10%), *Actinobacteria* (3%), and *Verrucomicrobia* (13). Gut microbiota plays an important role in human health through maintaining the integrity of the host intestinal epithelial barrier, digesting indigestible nutrients and providing nutrients, modulating the host innate immune system, and defending against pathogens (14). The gut microbiota colonizes and reproduces on intestinal epithelial surfaces, secreting enzymes that catalyze carbohydrate and metabolize indigestible carbohydrates into gut metabolites, vitamins, and some neurochemicals (15). Furthermore, homeostasis of gut microbiota positively affects host immuno-function and prevents invasion by pathogenic microorganisms (16). However, despite the fact that the intestinal microbiota is indispensable for maintaining lifelong health of humans, detailed understanding of the process is still at a juvenile stage. Fungi, protists, archaea, and viruses also belong to the gut microbiota, but less is known about their roles in gut homeostasis (17). Furthermore, most studies are based on snapshots of microbiome landscapes at only one time point and there is no general, effective, and systematic detection method in clinic practice.

Intestinal dysbiosis refers to imbalance in the composition and abundance of gut microbiota and is associated with numerous diseases and phenotypes (18). For healthy individuals, the gut microbiota is in dynamic equilibrium, depending mainly on genetic factors, nutrient availability, dietary habits, psychological states, etc. (19–21). Once the balance is aberrant, disorders in the composition and structure of the intestinal microbiota transform the intestinal microecological environment from healthy state to disease state, subsequently resulting in the pathogenesis of various common metabolic disorders including obesity, type 2 diabetes, non-alcoholic liver disease, cardio-metabolic diseases, and malnutrition (22, 23). CVD is one such disease, which is closely related to metabolic disorders and inflammation (24). Therefore, changes in gut microbial community structure and diversity are also one of the key factors leading to the onset and development of CVDs, as revealed by accumulating evidence (25). This review highlights the changes of certain gut microbes in patients with atherosclerosis, hypertension, heart failure, myocardial infarction, atrial fibrillation (AF), and chronic kidney disease (CKD), respectively (**Supplementary Table 1**). As the results shown, patients with different CVDs imply various responses in terms of gut microbial diversity, abundance, and homogeneity. For example, studies on the intestinal flora of patients with prehypertension and hypertension have found that the most significant characteristics are the reduction and uneven distribution of microbial diversity and abundance, mainly manifested in the increase of *Firmicutes*/*Bacteroidetes* ratio, the decrease of probiotics, and the increase of *Prevotella* and *Klebsiella* (26). This result has been confirmed by microbiota transplantation experiment.

## TMAO and CVDs

Gut microbiota-derived metabolites, such as short-chain fatty acids, BAs, catechin, and TMAO, are the direct factors causing CVDs, and have been viewed as a new breakthrough for the prevention and treatment of CVDs (27, 28). TMAO keeps increasing the risks of CVDs after excluding other risk factors for CVDs, demonstrating a strong association between high circulating levels of TMAO and the risk and mortality of CVDs (29, 30). Consequently, a high circulating level of TMAO is a potential marker to better stratify CVDs risks and predict short- and long-term major adverse cardiac events (31). Understanding the specific mechanisms of TMAO in the progression and pathogenesis of CVDs will facilitate the development of new therapeutic approaches, and holds great promise for precise intervention in the occurrence of CVDs (32).

## The gut microbiota and TMAO

The gut microbiota is an important regulator that influences cardiovascular function through the production of intestinal metabolites such as TMAO. The structure and function of the gut microbiota varies and is affected by dietary components, thus affecting the synthesis of TMAO. TMA is a precursor to TMAO, and its dietary precursors are generally choline, L-carnitine, and betaine (33). Dietary sources of choline are abundant and include eggs, fish, grains, milk, meat, and their derived food. Some vegetables, such as soybeans and potatoes, also contain a lesser amount of choline (34). L-carnitine is predominantly contained in red meat (35), while most betaine is found in fish, beetroot, and grains grown in high osmotic environments, such as beans (36). More information on dietary precursors of TMA is shown in **Table 1**.

**Table 1:** The dietary precursors of TMA.

Dietary components	Dietary source	References
Choline	Eggs, fish, seafood, liver, beef, fish, pork, chicken, grains, milk, dairy meat, poultry, soy products, soybeans, and potatoes	(37, 38)
Phosphatidylcholine	Eggs, dairy, and meat	(39)
Betaine	Shellfish, wheat, beets, spinach, fish, shrimp, wolfberry, and beans	(40, 41)
L-carnitine	Red meat, such as beef and lamb; fish, poultry, and milk	(35)

These dietary components are metabolized to TMA under the participation of gut microbiota. However, despite the gut microbiota being instrumental in this process, only a few gut microbes have genes that utilize dietary precursors. Rath et al. found that less than 1% of the microbes in stool samples encoded the choline-utilizing TMA lyase system (*CutC/D*) and the carnitine Rieske-type oxygenase/reductase system (*CntA/B*), which depends on the availability of molecular oxygen (42). *YeaW/X*, which is a homolog of *CntA/B*, can also metabolize carnitine, choline, γ-butylbetaine, and betaine to generate TMA (43). Identification of these enzymes has made it possible to determine which bacteria synthesize TMA by using whole-genome sequencing analysis. The genes *CntA/B* have been detected in the genus *Acinetobacter* (e.g. *Acinetobacter calcoaceticus*, *Acinetobacter baumannii*), while the *CutC/D* genes have been detected in the genus *Pelobacter* (e.g. *Pelobacter acetylenicus, Pelobacter carbinolicus*) (44). These bacteria grow in different conditions, as shown in **Supplementary Table 2**. Consequently, different dietary precursors are degraded by different gut bacteria. Rath et al. showed that carnivores and omnivores had more *CntA/B* and *CutC/D* genes in their gut bacteria compared with herbivores, and these genes were found in more than 80 percent of gut bacteria. Overall, potential TMA-forming bacteria adapted to various nutritional conditions, although they were not evenly distributed (42). Most TMA produced in the gut is absorbed into the blood intravenously and into the liver by portal vein circulation. The liver is the home of TMA processing. Flavin-containing monooxygenases (FMOs) are abundant in liver microparticles and oxidize TMA to TMAO. For this catalytic reaction, FMOs require the cofactor flavin adenine dinucleotide and depend on nicotinamide adenine dinucleotide phosphate or nicotinamide adenine dinucleotide as a coenzyme (45). The reaction involves inserting an oxygen atom from O_2_ into TMA, while the other oxygen atom is reduced to water (46). FMOs in the liver are crucial in the production of TMAO, and experiments showed that FMO3 knockdown mice had decreased circulating levels of TMAO (32). Ultimately, TMAO is distributed throughout the body, being eliminated *via* the kidneys or accumulating in tissues (47).

**Figure 1:**
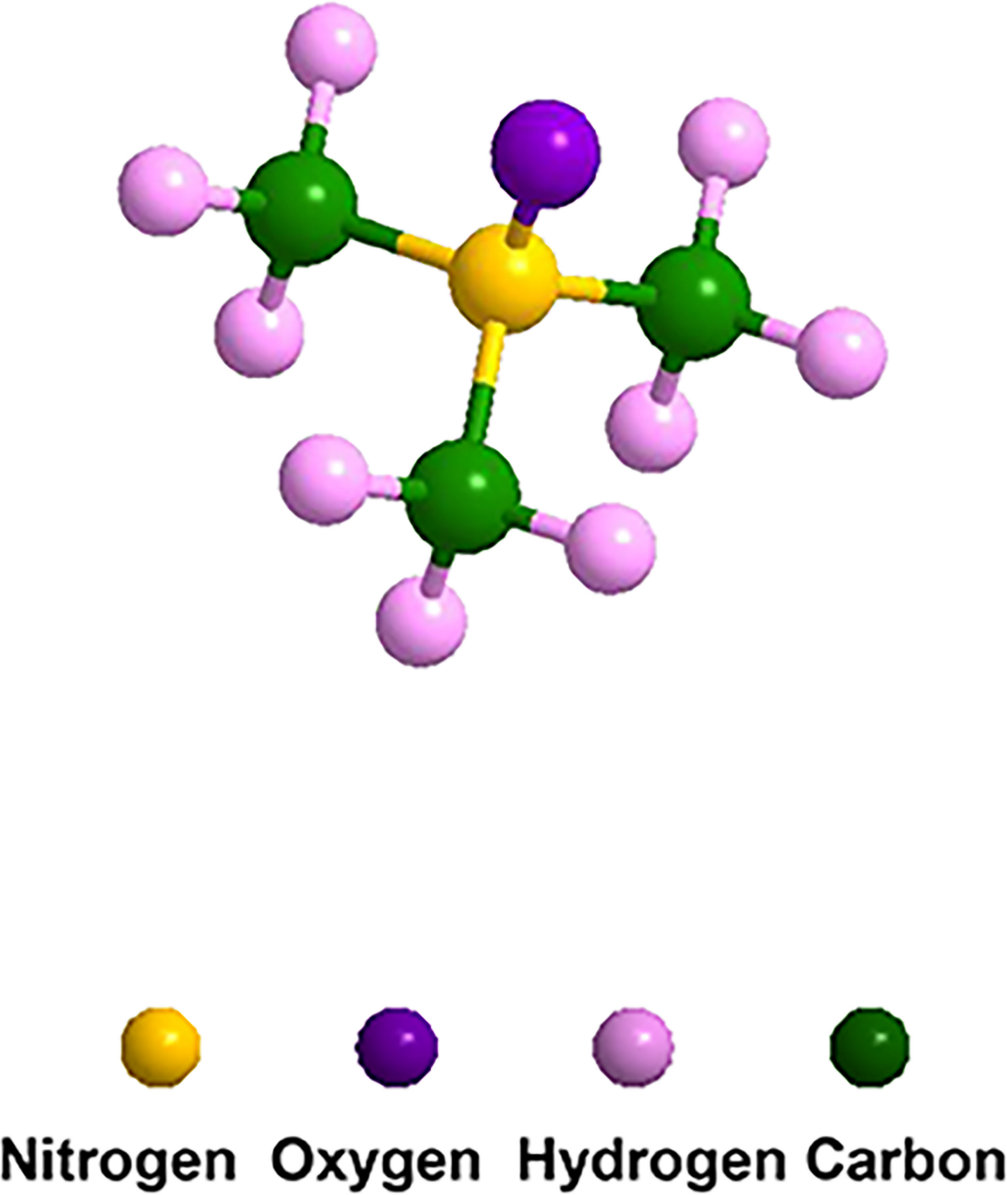
Molecular structure of TMAO. The formula is C_3_H_9_NO, which consists of three carbon atoms, nine hydrogen atoms, one nitrogen atom and one oxygen atom.

TMA is a volatile gas that smells fishy, while TMAO is a less volatile solid with the formula (CH_3_)_3_ NO (48), as shown in **Figure 1**. TMAO has a high turnover rate, with more than 90% excreted in the urine after being metabolized by the kidney (49). A small amount of TMAO is excreted by respiration and feces, and the rest is reduced to TMA by TMAO reductase and re-enters the circulation. TMAO-an important stabilizer of protein and nucleic acid-stabilizes proteins by modifying their polarity, and indirectly affects the balance between unfolded and folded conformations (50). The physiological functions of TMAO in the host are determined by these features, such as influencing the protein unfolding or endoplasmic reticulum stress response in cells (51).

**Figure 2:**
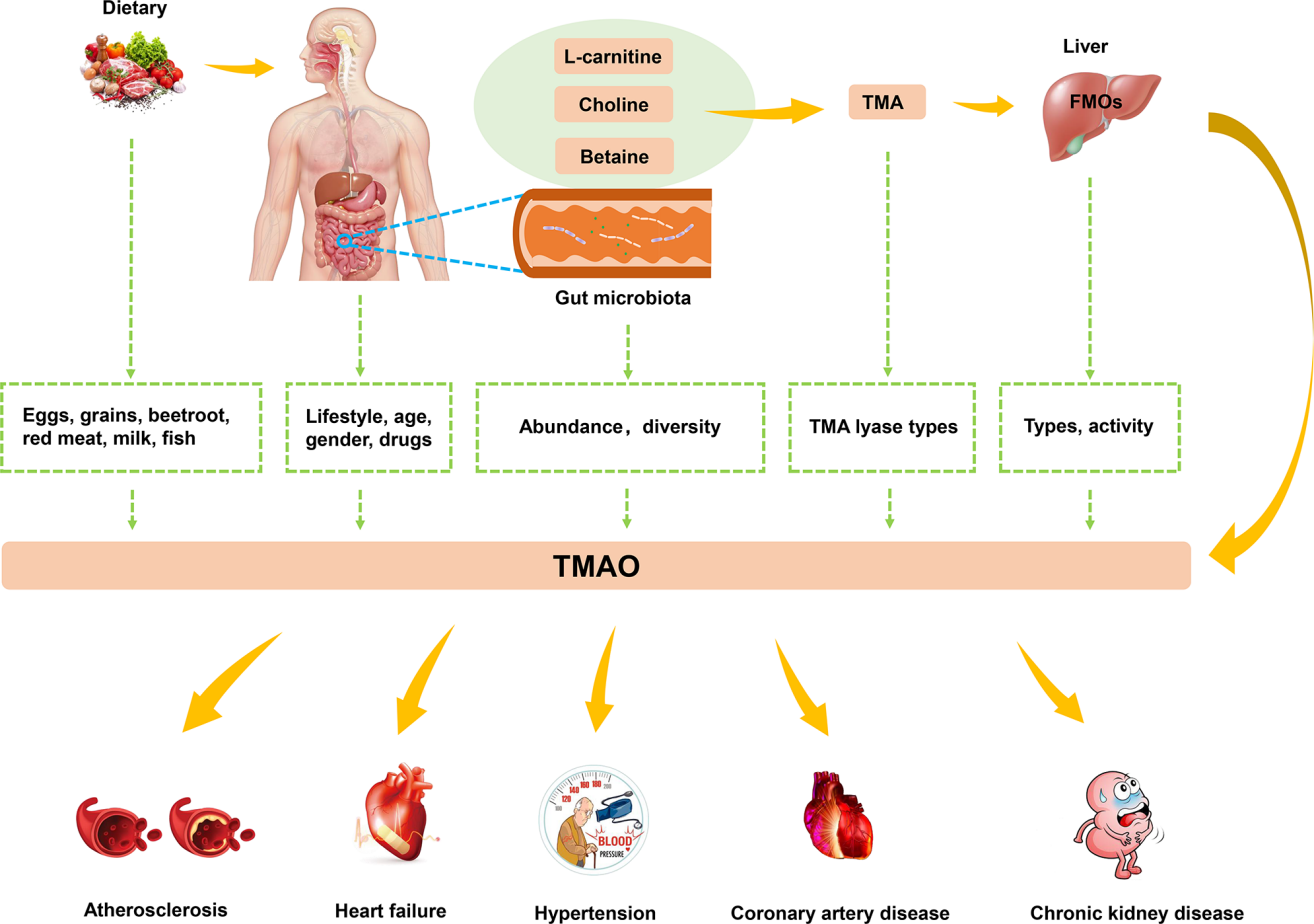
The biogenesis and metabolism of TMAO. Dietary choline or L-carnitine in food was metabolized to TMA by gut microbiota, which converted to TMAO by FMO3 in the liver. TMAO was closely related to the development of atherosclerosis, HF, hypertension and other CVDs.

Studies have found that high levels of TMAO are closely related to the occurrence of atherosclerosis, HF, hypertension, and other CVDs (**Figure 2**). In this context, lifestyle, gender, drugs, age, and microbiota have a synergistic impact on the levels of TMAO (52). Dietary pattern plays a crucial role in human microbiota (53). Western diets are characterized by animal products containing large amounts of TMA precursors, and not only affect the gut microbiota but also increase levels of TMAO (54). Exercise also affects the integrity of the gut and the intestinal microbiota, and was found to effectively decrease TMAO levels (55). Considering the characteristics of TMAO formation, the activity of FMOs also affects TMAO levels. FMO3 has the highest activity among all types of FMOs capable of oxidizing TMA to TMAO. Males were reported to have lower expression levels of FMO3 compared with females, which resulted in differences in TMAO levels (56). The gender specificity in TMAO levels has been demonstrated in the livers of mice, but no difference in TMAO levels has been detected between human men and women (57). Drugs have been shown to have an impact on the human gut microbiota (58). For example, in human experiments conducted by Tang et al., TMAO plasma levels were markedly decreased after administration of antibiotics but returned to the previous level after withdrawal of antibiotics (59). In addition, plasma TMAO levels have been reported to gradually increase with age, and this process is regulated by changes in diet and an increase in abundance of TMA-producing bacteria (60).

## The mechanism of TMAO leading to CVDs

The mechanism of TMAO resulting in atherosclerosis, HF, and hypertension has gradually been clarified (**Supplementary Table 3**).

## TMAO and atherosclerosis

**Figure 3:**
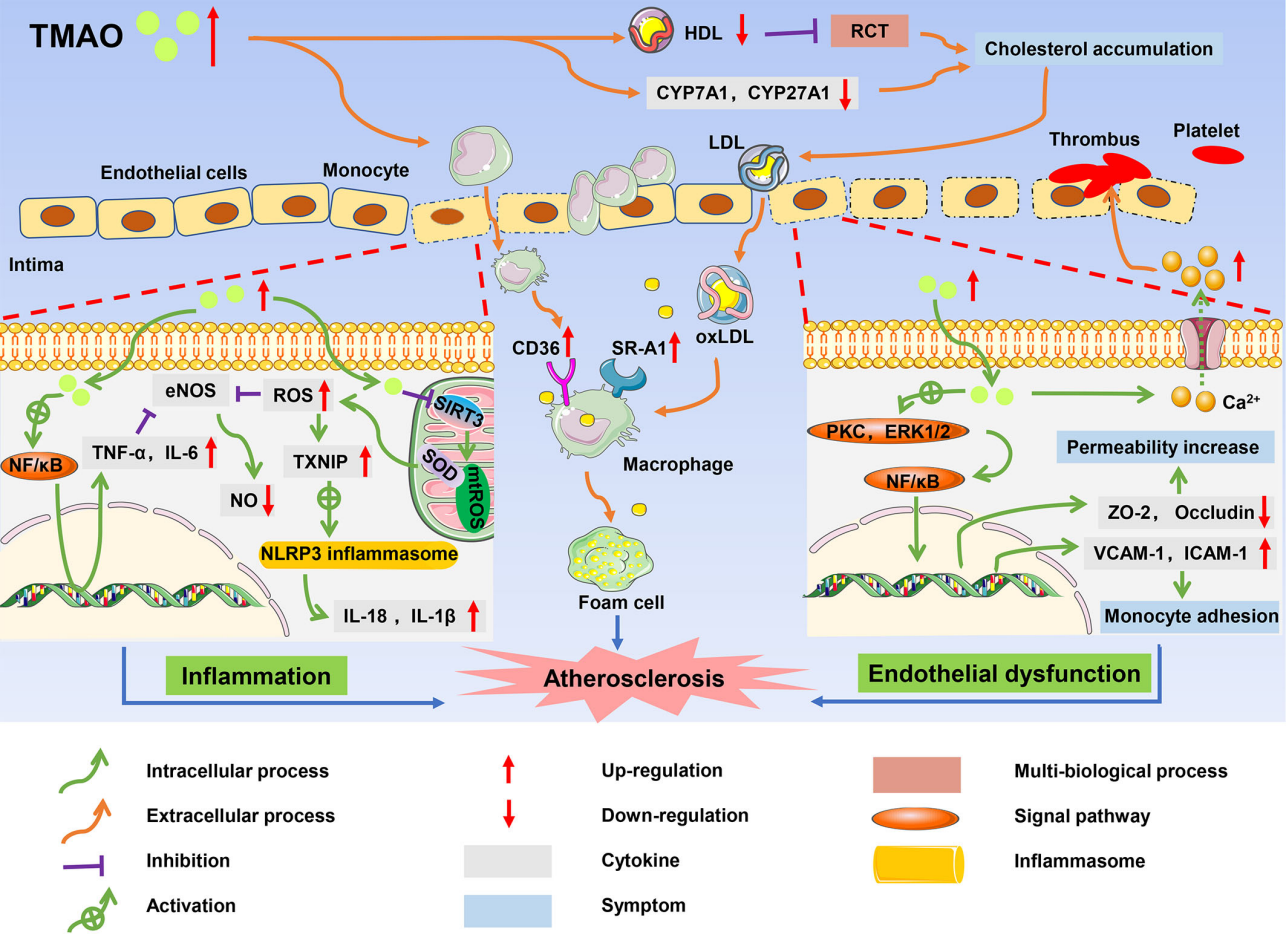
The role of TMAO in onset and progression of atherosclerosis. High circulating levels of TMAO inhibited RCT, and accumulated cholesterol, which promoted the formation of foam cells; TMAO induced inflammatory responses, resulting in increased production of pro-inflammatory cytokines such as TNF-α and IL-1B; TMAO caused endothelial dysfunction and platelet activation, and ultimately contributed to the development of atherosclerosis.

Atherosclerosis, which is the major risk factor of CVDs, is characterized by foam cell formation, inflammatory cell recruitment, and endothelial dysfunction (61). A high level of TMAO is thought to contribute to atherosclerosis, leading to a long-term risk of CVDs and poor prognosis in patients (32). The mechanism by which TMAO promotes the complex pathological processes of atherosclerosis is gradually being elucidated and includes cholesterol accumulation, activation of pro-inflammatory pathways, endothelial dysfunction, and thrombosis (62) (**Figure 3**).

### Inhibition of cholesterol metabolism and stimulation of foam cell formation

There are multiple ways in which TMAO affects cholesterol metabolism and promotes the formation of foam cells. The reverse cholesterol transport (RCT) pathway involving high-density lipoprotein (HDL) is an important process to maintain the balance of cholesterol metabolism, and promotes the efflux of free cholesterol from macrophages (63). Studies found that HDL in the plasma of CVD patients with high TMAO levels was significantly reduced, resulting in inhibition of the RCT pathway and cholesterol accumulation in macrophages (64). TMAO also upregulated the expression of scavenger receptor cluster of differentiation 36 (CD36) and scavenger receptor-A1 (SR-A1) in macrophages, and induced cholesterol flow to macrophages. Wang et al. measured the contents of several scavenger receptors of macrophages in ApoE^-/-^ mice and found that CD36 and SRA1 levels were increased in macrophages in TMAO-treated mice compared with control mice (65). Under the interference of these factors, cholesterol-overloaded macrophages developed into foam cells, which is one of the earliest cellular hallmarks of atherosclerosis (66). Furthermore, TMAO is closely related to BA metabolism, negatively impacting the main pathway of cholesterol elimination in humans. Cholesterol is concentrated in the liver by the RCT pathway and then secreted into the bile in the form of unesterified cholesterol by the ATP-binding cassette (ABC) transporters G5 (ABCG5) and G8 (ABCG8) (67). Alternatively, cholesterol can be synthesized as BAs, which are then transported to bile *via* a bile salt export pump (BSEP) and finally excreted with feces (68). In addition to the hepatic cholesterol excretion pathway, the ABG5/G8 heterodimer promotes cholesterol excretion from the intestinal cells directly to the intestinal lumen. In C57BL/6J mice, TMAO significantly decreased the expression of ABCG5/G8 (69). Moreover, TMAO activated the farnesoid X receptor (FXR) and small heterodimer partner (SHP), downregulating the expression of hepatic BA synthase cytochrome P450 family 7 subfamily A member 1 (CYP7A1), cytochrome P450 family 27 subfamily A member 1 (CYP27A1), and BA transporter organic anion-transporting polypeptide (OATP) in ApoE^-/-^ mice (70). Inhibition of the related enzymes reduced cholesterol elimination, causing the accumulation of cholesterol in the liver. Cholesterol then re-entered the blood through the low-density lipoprotein (LDL) cycle, which increased foam cell formation and accelerated the process of atherosclerosis.

### Inflammatory response

Atherosclerosis is also considered a chronic inflammatory disease because the endothelial inflammatory damage is ongoing throughout atherosclerosis progression (71). High TMAO levels were found to induce activation of the nuclear factor-kappa B (NF-κB) pathway and increase the expression of proinflammatory genes including those encoding inflammatory cytokines, adhesion molecules, and chemokines (72). Similar results were reported in the study of Chen et al., where high TMAO levels increased the expression of tumor necrosis factor-α (TNF-α) and interleukin-6 (IL-6), but inhibited the expression of anti-inflammatory cytokine interleukin-10 (IL-10), thereby accelerating cellular inflammation in obese mice treated with a Western diet (73). Furthermore, *in vitro* studies of cultured endothelial progenitor cells (EPCs) by Chou et al. showed that TMAO not only promoted cellular inflammation but also accounted for oxidative stress, thus playing an indirect role in promoting expression of inflammatory cytokines (74).

In human umbilical vein endothelial cells (HUVECs) and aortas from ApoE^-/-^ mice, TMAO increased the concentrations of reactive oxygen species (ROS), especially superoxide, by inhibiting the SIRT3-SOD2-mitochondrial ROS signaling pathway (75). The increase of ROS and TMAO-derived oxidative stress-related molecule malondialdehyde (MDA) impaired the activity of endothelial nitric oxide synthase (eNOS), contributing to decreased nitric oxide (NO) bioavailability and endothelial dysfunction in aged rats (76, 77). In addition, thioredoxin-interacting protein (TXNIP) is an important protein that mediates ROS and the NOD-like receptor protein 3 (NLRP3) inflammasome (78). Upregulation of TXNIP by ROS stimulated the NLRP3 inflammasome, which activated the protease caspase-1 to facilitate the release of inflammatory cytokines such as interleukin-18 (IL-18) and interleukin-1β (IL-1β) and triggered inflammatory responses. Activated inflammatory factors also aggravated the damage caused by oxidative stress in a positive feedback manner. For example, TNF-α and IL-6 reduced the production of NO by inhibiting the phosphorylation of eNOS (79). Oxidative stress and inflammation reinforced each other in a continuous cycle that accelerated atherosclerosis (80).

### Endothelial dysfunction and thrombosis

TMAO may lead to endothelial dysfunction. Endothelial tight junction proteins play a barrier role in regulating cell permeability and maintaining cell polarity (81). TMAO activated the expression of the inflammatory mediator high mobility group box 1 (HMGB1), which stimulated multiple receptors such as toll-like receptor 4 (TLR4), thus mediating extracellular signal-regulated kinases 1 and 2 (ERK1 and ERK2) and the NF-κB signaling pathway (82). Activation of this pathway destroyed the expression of tight junction proteins such as zonula occludens-2 (ZO-2), occludin, and vascular endothelial cadherin (VE-cadherin) in endothelial cells, which increased endothelial cell permeability. Gaps between the endothelial cells allowed LDL to easily enter the intima and be oxidized to oxidized low-density lipoprotein (ox-LDL) (83). In addition, TMAO also activated proteinase C, and induced functional damage of vascular endothelial cells through the PKC/NF-κB pathway. With the induction of the NF-κB signaling pathway in HUVECs, the subsequent upregulation of expression of vascular cell adhesion molecule-1 (VCAM-1) and intercellular adhesion molecule-1 (ICAM-1) enhanced the adhesion of monocytes to the vascular cells (84). Activated monocytes were recruited to the intima and differentiated into macrophages, accelerating endothelial dysfunction (85). In addition, TMAO inhibited endothelial cell proliferation and impaired endothelial self-repairment (86). Platelet hyperreactivity and thrombosis are also associated with TMAO (87). According to experiments by Zhu et al., TMAO improved the expression of adenosine diphosphate (ADP) and thrombin to enhance a release of Ca^2+^ from intracellular calcium stores, causing platelet aggregation and enhancing thrombosis potential (88).

TMAO, as one of the independent risk factors of CVDs, is widely distributed in human tissues and body fluids such as urine, blood, feces, bile, and semen. Once TMAO formation/concentration is elevated, it may increase the risks of atherosclerosis for healthy individuals (48). However, owing to the complexity of individual physiological characteristics, the threshold concentration of TMAO that causes atherosclerosis is not clear.

## TMAO and HF

**Figure 4:**
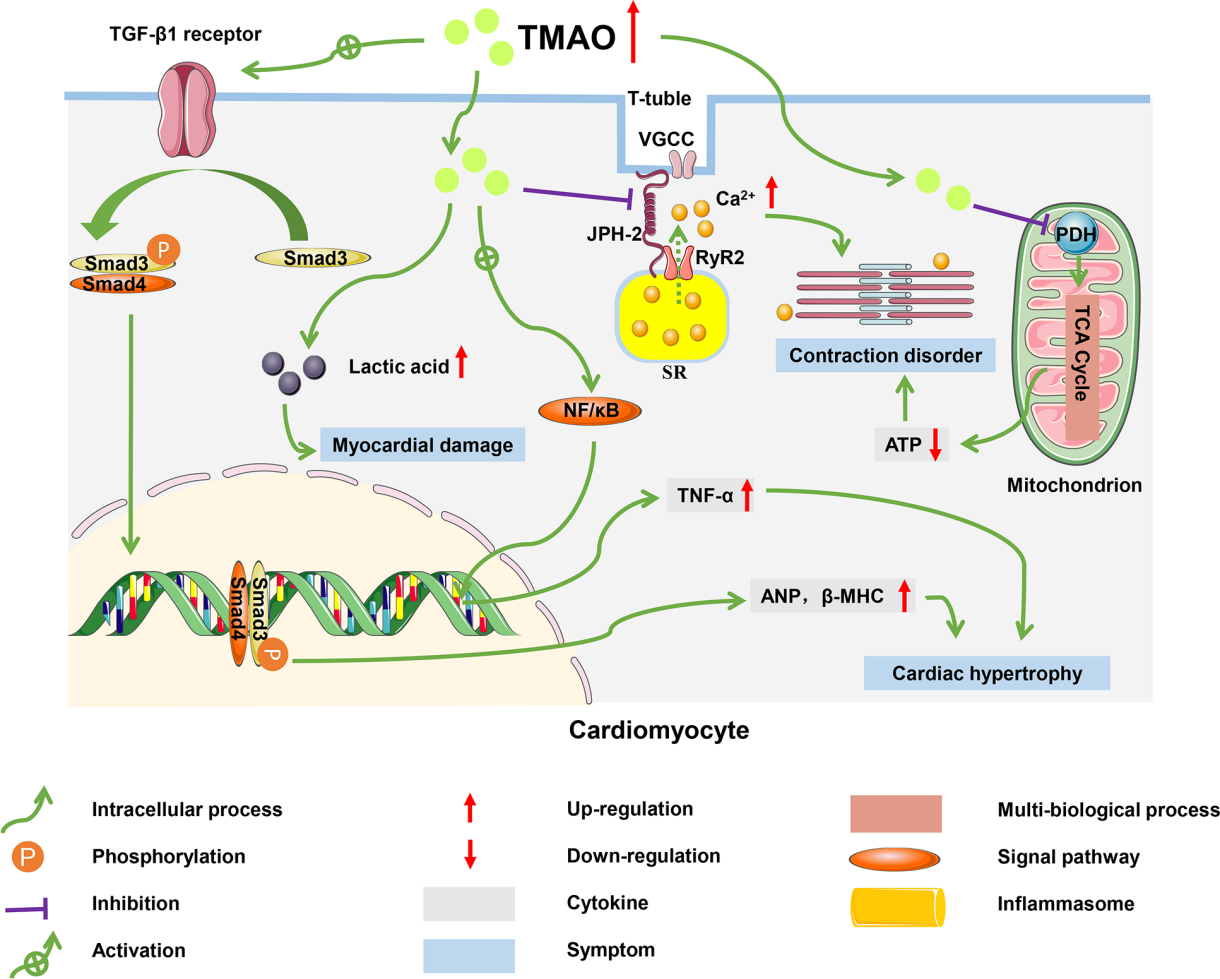
Molecular mechanisms of HF progression regulated by TMAO. In cardiomyocytes, TMAO activated TGF-β1/Smad3 pathway and increased expression levels of ANP and β-MHC, which caused myocardial hypertrophy and fibrosis; TMAO decreased energy metabolism and mitochondrial function by inhibiting the activity of pyruvate, leading to myocardial damage; TMAO also negatively affected the intracellular Ca^2+^ processing and myocardial contraction.

HF is defined as a group of syndromes caused by structural or functional damage that results in impaired ventricular filling or cardiac output, and which involves multi-organ and systemic neurohumoral dysfunction (89). As the terminal stage of various CVDs, HF is associated with a persistently high rate of morbidity, hospital readmission, and mortality in patients and has become a major public health problem worldwide, with more than 60 million cases diagnosed globally (47). Although there is an active search for effective treatments of HF, up to 50% of diagnosed patients have died in the past 30 years, indicating that the pathogenesis of this disease has not yet been fully elucidated (90). Accumulating evidence has indicated that TMAO is involved in the progression of HF (91). A clinical study in patients with HF revealed that plasma TMAO levels were positively associated with the risk of chronic HF, suggesting that TMAO might be a new target for the treatment of HF (49, 92). In addition to inducing HF by promoting atherosclerotic progression, elevated plasma TMAO levels may accelerate myocardial hypertrophy, exacerbate mitochondrial dysfunction, and induce transverse-tubule (T-tubule) remodeling in cardiomyocytes (47, 93). These pathophysiological mechanisms have the potential to form a vicious circle that further aggravates the development of HF (**Figure 4**).

### Myocardial hypertrophy and fibrosis

TMAO significantly increased the mRNA and protein levels of the hypertrophic markers atrial natriuretic peptide (ANP) and β-myosin heavy chain (β-MHC), causing myocardial hypertrophy and fibrosis (94). In mice fed a high choline diet, myocardial hypertrophy induced by TMAO was achieved through the involvement of transforming growth factor-β1 (TGF-β1) and small mothers against decapentaplegic (Smad), an intermediate effector molecule in the TGF-β superfamily signaling pathway (95). High levels of TMAO activated the TGF-β1 receptor, and then Smad3 was phosphorylated at its C-terminus by the TGF-β1 receptor. Subsequently, phosphorylated Smad3 bound to Smad4 (the partner Smad) and was transported from the cytoplasm to the nucleus to activate the transcription of downstream target genes related to ANP and β-MHC (96). In addition, TMAO induced myocardial inflammation, which may lead to myocardial fibrosis and cardiac dysfunction in western diet-induced obese mice (97). Zhang et al. found that mice fed on diets supplemented with high sugar and fat had elevated plasma TMAO levels, accompanied with increased TNF-α levels and decreased IL-10 levels, which led to myocardial fibrosis (98).

### Exacerbation of mitochondrial dysfunction

The accumulation of TMAO in cardiac tissue led to disturbed energy metabolism and impaired myocardial function (99). Savi et al. found that TMAO induced mitochondrial dysfunction, thereby decreasing contractility of cardiomyocytes (100). Mitochondria in cardiomyocytes consume pyruvate and fatty acids to generate energy, which maintains the physiological function of the heart. However, TMAO inhibited the activity of pyruvate dehydrogenase (PDH) and weakened the oxidation of pyruvate in mitochondria, which impaired production of ATP (101). The decrease in ATP resulted in a decline in contractile capacity of the cardiomyocytes. TMAO also caused glycogen accumulation and lipofuscin-like pigment deposition in the paranuclear area of cardiomyocytes, which generated damage to mitochondria, the proteasome system, and lysosomal function (102).

### T-tubule remodeling in cardiomyocytes

TMAO disturbed myocardial excitation-contraction coupling by inducing Ca^2+^ dysfunction (103). Microtubules formed by the polymerization of α- and β-tubulin dimers are ubiquitous cytoskeletal fibers in cells and are instrumental in maintaining ventricular shape (104). In a HF model with coarctation of the aorta, mice fed a high-cholesterol diet had higher TMAO levels and accelerated adverse ventricular remodeling (105). Jin et al. reported the same conclusion, with TMAO promoting tubulin densification and increased polymerization and redistribution of proteins such as Junctophilin-2 (JPH2). The translocated JPH2 spanned T-tubule and sarcoplasmic reticulum (SR) membranes and enabled T-tubule remodeling in cardiomyocytes (106, 107). The remodeling of T-tubules destroys accurate communication between L-type voltage-gated Ca^2+^ channels (VGCCs), located predominantly on the T-tubule membrane, and type 2 ryanodine receptor (RyR2), a Ca^2+^-release channel located on the sarcoplasmic reticulum (108). This disrupted communication leads to a massive efflux of Ca^2+^. The Ca^2+^ dysfunction impairs the excitation-contraction coupling of the myocardium and worsens HF.

### Indirect effects of renal function

In addition to myocardial injury, TMAO indirectly affects the progression of HF by influencing renal function (109). Tang et al. reported that a high level of TMAO increased Smad3 phosphorylation, which promoted renal interstitial fibrosis and dysfunction. Consequently, sodium and water were retained in the kidneys, which increased cardiac preload and exacerbated HF (110).

Based on the role of TMAO in the progression of HF, regulating TMAO levels is a new and attractive therapeutic target for HF treatment (111). However, considering the diversity of gut microbiota and TMAO levels in patients with HF, specific treatment strategies need to be developed.

## TMAO and hypertension

**Figure 5:**
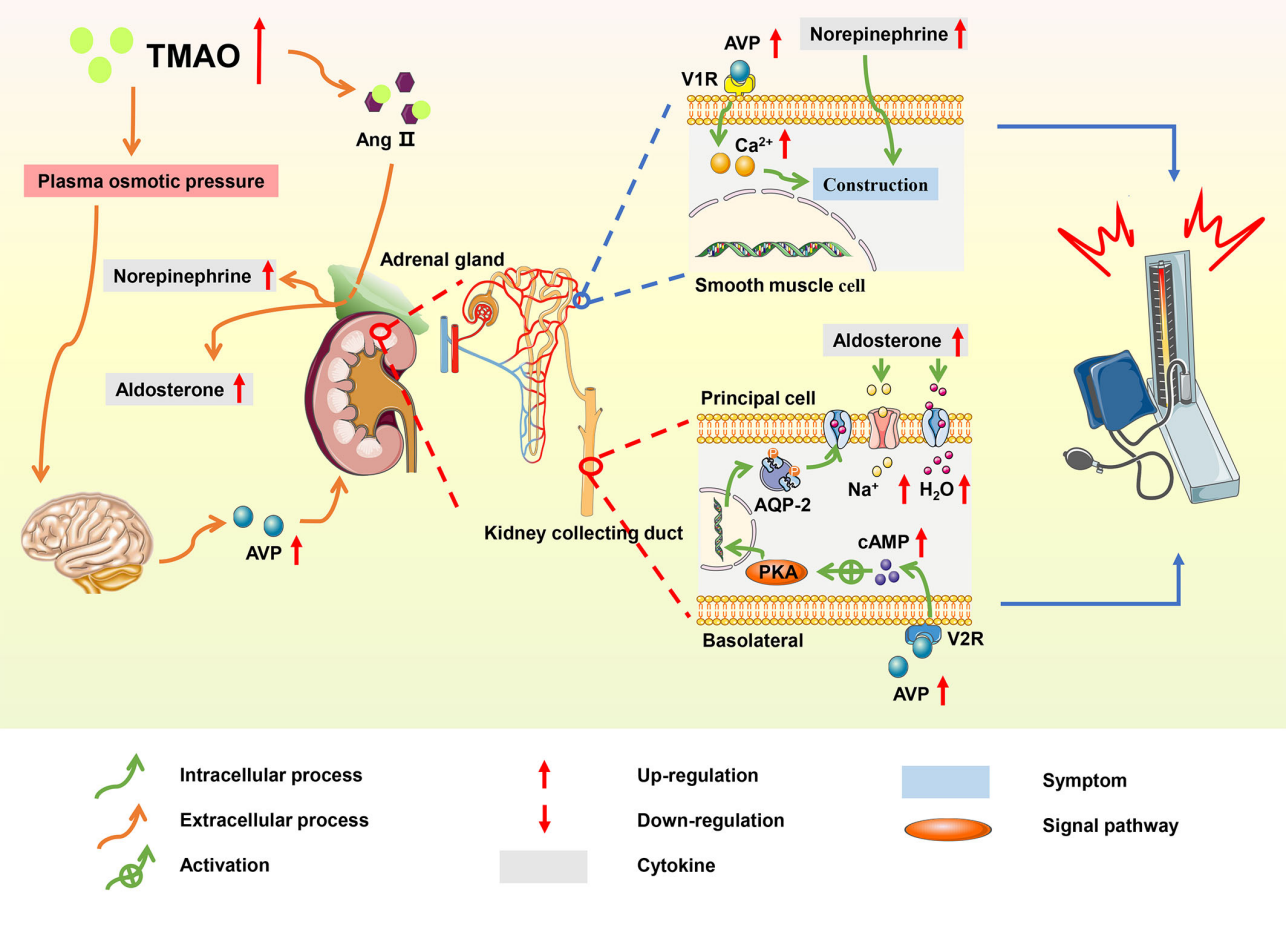
Summary schemes illustrating the involvement of TMAO in hypertension. TMAO prolonged the hypertensive effect of Ang II, and elevated blood pressure *via* RAAS; TMAO stimulated the secretion of AVP, increasing water reabsorption through the ‘TMAO-AVP-AQP-2 Axis’, which contributed to the progression of hypertension.

By 2025, the total number of patients with hypertension is expected to reach 1.56 billion and the average age of patients tends to be younger (112). More than 95% of hypertension is related to lifestyle, hence a healthy lifestyle tends to better control the onset and development of CVDs (113). Several dietary interventions, such as the Mediterranean diet (Med-Diet) and the Dietary Approaches to Stop Hypertension (DASH) diet, have shown that a high intake of fruits, vegetables, and fiber is conducive to alleviating hypertension (114). However, there are other factors involved in the development of hypertension, and studies have found a link between the gut microbiome, microbial metabolites, and hypertension (115). In the hypertensive rat model, Yang et al. found that microbial abundance and diversity were reduced (116). Meanwhile, a meta-analysis of population-based studies found that individuals with high TMAO levels were more likely to develop hypertension compared with those with low TMAO levels (117). TMAO prolongs the hypertensive effect of angiotensin II (Ang II) and increases water reabsorption and has emerged as a potential biomarker of hypertension (**Figure 5**).

### An extension of the enhancement of Ang II

Ang II is the main effector of the renin-angiotensin-aldosterone system (RAAS), and studies have shown that Ang II promoted the development of hypertension through RAAS system (118). In hypertensive mice induced by Ang II, TMAO changed the structure of Ang II by enhancing folding and processing of proteins, thereby prolonging the duration of hypertensive effect (119). In the adrenal medulla, Ang II promoted the synthesis of norepinephrine, which contracted the vascular arteriolar smooth muscle and increased blood pressure; meanwhile, in the adrenal cortex, Ang II stimulated the secretion of aldosterone, thereby increasing the absorption of water and sodium in collecting duct principal cells (120).

### Increase in water reabsorption

In a spontaneous hypertensive rat model, TMAO increased plasma osmotic pressure and water reabsorption *via* the ‘TMAO-AVP-(AQP-2) axis’, which contributed to hypertension (121). High TMAO levels promoted the elevation of plasma osmotic pressure, increasing the secretion of arginine vasopressin (AVP) from the posterior pituitary. The effects of AVP were initiated through the binding of AVP to the type-2 vasopressin receptor (V2R) on the basolateral membrane of principal cells in the kidney collecting duct (122). This binding activated cyclic adenosine monophosphate/protein kinase A (cAMP/PKA) signaling pathway, resulting in the translocation of aquaporin-2 (AQP2) from the vesicle to the apical plasma membrane and markedly increasing the water reabsorption of principal cells (123). AVP also stimulated the type-1 vasopressin receptor (V1R) of vascular smooth muscle to cause vasoconstriction and increase blood pressure. Additionally, TMAO induced hypertension by accelerating vascular endothelial dysfunction (124).

## TMAO and arrhythmia

Arrhythmia, which primarily includes AF, ventricular arrhythmia (VA), and atrioventricular block, is emerging as an intractable CVD that leads to HF or sudden cardiac death (62). Meng et al. first proposed that TMAO may be a target to treat arrhythmia (125). A study in a community cohort of elderly participants demonstrated that there was a positive association between plasma TMAO levels and long-term incident AF in patients with suspected stable angina (126). TMAO also promoted the development of VA in various ways and several of these reports highlighted ischemia-induced VA. Gong et al. found that TMAO promoted the release of pro-inflammatory cytokines such as IL-1β and TNF-α to stimulate the cardiac autonomic nervous system (CANS) and worsen ischemia-induced VA (127). In addition, TMAO significantly promoted ischemic-induced VA by modifying the left stellate ganglion (LSG) either directly or through the ‘gut-brain-heart’ axis (125).

## TMAO and coronary artery disease

CAD is a CVD with the highest morbidity and mortality in the world, and is closely related to intestinal microbiota dysbiosis (128). According to the research, gut microbiota may be an important diagnostic marker for CAD in the future (129). As an active metabolite synthesized by intestinal microbiota, the increased level of TMAO is associated with increased risks of CAD and all-cause mortality (130, 131). In addition, Guo et al. reported that associations of TMAO with CAD were sex-related (132). However, although TMAO may be a potential target for the prevention and treatment of CAD, current immature conclusions limit clinical applications at present.

## TMAO and CVD-related diseases

In addition to CVDs, TMAO promotes numerous related diseases, such as diabetes, CKD, etc., which have a burden of cardiovascular complications (133, 134). Li et al. showed that elevation of plasma TMAO increased the chance of developing gestational diabetes by 22% (135). TMAO stimulated gluconeogenesis and caused insulin resistance, which promoted the progression of diabetes (136). As a major risk factor for CVDs and cardiovascular complications, the relationship between plasma TMAO concentrations and the increased risks of CVDs in individuals with type 1 and 2 diabetes has been reported (137–139). According to a study by Winther et al., higher concentrations of plasma TMAO were related to mortality, CVDs, and poor renal outcome in patients with type 1 diabetes (137). Similar results were reported in the clinical study of patients with type 2 diabetes, where increased TMAO levels are independently associated with risks of adverse cardiovascular events (138, 139). Furthermore, some studies on the relationship between TMAO levels and CVDs identified kidney function as an important confounder, owing to a degree of decline in the renal function of patients with CVDs (140). Elevated TMAO levels damaged renal medulla cells and influenced glomerular filtration, which caused water and sodium retention and impaired the kidneys (30). Another study suggested that plasma TMAO levels were inversely correlated with long-term survival in patients with CKD (141). Despite these positive findings, additional research is needed to clarify the specific mechanism of TMAO involvement in diseases related to CVDs.

## Strategies to regulate TMAO/TMA

Increasing numbers of experiments that are aimed at altering host gut microbiota and interfering with the TMAO synthesis pathway as therapeutic means have been conducted to prevent and treat CVDs. Considering the multifactorial nature of TMAO generation, many strategies can be pursued to reduce the level of TMA/TMAO in patients with CVDs, such as lifestyle changes, use of antibiotics and functional foods, development of related inhibitors, and improvement of gut microbiota (**Supplementary Table 4**).

### Changing eating behavior is an effective approach

Choline, phosphatidylcholine, and L-carnitine are the precursors of TMA and are found in high levels in the daily diet, especially in red meat (142). Reducing the intake of red meat can theoretically reduce the level of TMA in the body, however, there is no universal international control standard. Besides, a vegetarian lifestyle, with multiple health benefits, also reduces the risk of CVDs (143).

### Antibiotics use is another strategy

Numerous studies have shown that the use of antibiotics can significantly reduce circulating TMAO levels, possibly due to changes in the gut microbiota composition. However, the use of antibiotics also poses concerns, such as transient changes in circulating TMAO levels, increased risk of bacterial resistance, and other antibiotic safety problems (144). According to one study, older women who took antibiotics for years were prone to inflammation caused by intestinal microbiota translocation (145). Therefore, the use of antibiotics needs caution.

### The development of related inhibitors also plays a role in TMAO regulation

Animal experiments showed that inhibition of choline TMA lyase by 3,3-dimethyl-1-butanol (DMB) could reduce plasma TMAO levels and aortic lesions in mice; however, human clinical experiments have not been performed (146). Another blocking method is to inhibit hepatic FMO3 activity. Chen et al. found that using 3,3′-diindolyl methane or indole-3-carbinol to inhibit FMO3 could be a suitable target to control TMAO production (147).

### The use of probiotics to regulate gut microbiota is promising

Probiotics are beneficial living microbes and represent a great way to improve gut microbiota. In contrast to antibiotics, probiotics are inexpensive, noninvasive, and have few side effects. Multiple studies have shown the positive effects of probiotics in reducing TMA levels and alleviating CVDs, with the probiotics including *Enterobacter aerogenes*, *Lactobacillus rhamnosus*, *Saccharomyces boulardii*, and *Bifidobacterium animalis subsp*. *lactis* (148–151). Probiotics present an easy way to reduce possible cardiovascular risks and further studies are underway to identify more microbial strains with such benefits.

### Prebiotics can also be used to modulate gut microbiota

Prebiotics include galactooligosaccharides, fructooligosaccharides, inulin, dietary fiber, etc. that are utilized by host microbes (152, 153). Prebiotics can promote the growth of beneficial bacteria in the gut and decrease bacteria able to transform precursors into TMA. For example, mice fed resveratrol had remodeled gut microbiota and decreased production of TMA (154).

### Fecal microbiota transplantation is a popular approach

FMT was successfully applied in animal models and restored the structure of the gut microbiota in mice (155). However, an experiment on humans showed that FMT normalized insulin sensitivity of obese subjects with metabolic syndrome, but the effects were short-term (156). FMT has great potential as a treatment for CVDs, and further studies are needed to increase its efficacy and safety in reducing the risks of CVDs (157).

## Outlook

In conclusion, high concentrations of TMAO are associated with the development of various CVDs, such as atherosclerosis, hypertension, HF, arrhythmia, and CAD. However, although there are many reports on the harmful effects of TMAO, there are still some studies suggesting that TMAO plays a beneficial role. One experiment suggested that TMAO enhanced the stability of cellular proteins, which protected cells against the deleterious effects of various stressors. Furthermore, TMAO alleviated the formation of aortic atherosclerotic lesions in ApoE^-/-^ mice. Therefore, additional research is needed to determine the role of TMAO in CVDs.

CVDs remain the leading cause of death globally, despite the availability of numerous clinical treatments for CVDs. Gut microbiota and TMAO affect CVDs in various ways and interact with each other, and this link provides an opportunity to identify TMAO as a new therapeutic target to treat CVDs. Many studies have modulated the diversity of gut microbiota and TMAO/TMA levels in patients through dietary intervention, probiotics, and FMT. These strategies regulate gut microbial structure, reduce TMAO levels, and reduce the risk of CVDs. However, the complex composition of gut microbiota means that the gut microbiota that contributes most to CVDs and the specific mechanism of TMAO are not fully understood. Current understanding is predominantly based on animal experiments. Consequently, longitudinal studies in humans are needed to determine the role of gut microbiota and TMAO in CVDs and facilitate the development of better therapeutic strategies.

## Author contributions

JZ, ZZ, MH and H-XH wrote the original draft. E-HL, P-BW, XL, Y-TW, X-CC, J-QT and M-YZ revised the manuscript. LX and X-XK conceived the manuscript. All authors agreed to be accountable for the content of the work. All authors contributed to the article and approved the submitted version.
