# Supplementary material for: The gut microbial metabolite trimethylamine N-oxide and cardiovascular diseases
Source: Front Endocrinol (Lausanne). 2023 Feb 7;14:1085041. doi: 10.3389/fendo.2023.1085041 (PMC9941174; doi:10.3389/fendo.2023.1085041)
Supplement: Supplementary file 1 [file Table_1.docx]

**Supplementary Table 1.** Gut microbiota changes in patients with cardiovascular diseases

| Cardiovascular diseases | Family | Genus | Species | Increase/  decrease | References |
| --- | --- | --- | --- | --- | --- |
| Atherosclerosis | *Coriobacteriaceae* | *Collinsella* |  | Increase | (1,2) |
|  | *Eggerthellaceae* | *Eggerthella* | *E.lenta* | Increase |  |
|  | *Enterobacteriaceae* | *Escherichia* | *E.coli* | Increase |  |
|  |  | *Klebsiella* |  | Increase |  |
|  |  | *Enterobacter* | *E.aerogenes* | Increase |  |
|  | *Ruminococcaceae* | *Ruminococcus* | *R.gnavus* | Increase |  |
|  | *Eubacteriaceae* | *Eubacterium* |  | Decrease |  |
|  | *Lachnospiraceae* | *Roseburia* | *R.intestinalis* | Decrease |  |
|  | *Oscillospiraceae* | *Faecalibacterium* | *F.cf.prausnitzii* | Decrease |  |
|  |  |  | *F.prausnitzii* | Decrease |  |
| Hypertension | *Actinomycetaceae* | *Actinomyces* |  | Increase | (3,4,5) |
|  | *Clostridiaceae* | *Clostridium* |  | Increase |  |
|  | *Eggerthellaceae* | *Eggerthella* |  | Increase |  |
|  | *Enterobacteriaceae* | *Klebsiella* | *K. pneumoniae,* | Increase |  |
|  |  | *Salmonella* |  | Increase |  |
|  | *Eubacteriaceae* | *Eubacterium* | *E.siraeum* | Increase |  |
|  | *Porphyromonadaceae* | *Porphyromonas* |  | Increase |  |
|  | *Prevotellaceae* | *Prevotella* |  | Increase |  |
|  | *Rikenellaceae* | *Alistipes* | *A.finegoldii* | Increase |  |
|  | *Ruminococcaceae* | *Ruminococcus* | *R.torques* | Increase |  |
|  | *Streptococcaceae* | *Streptococcus* | *S.infantarius,* | Increase |  |
|  |  |  | *S.pasteurianus* | Increase |  |
|  |  |  | *S.salivarius* | Increase |  |
|  | *Tannerellaceae* | *Parabacteroides* | *P.merdae* | Increase |  |
|  | *Bacteroidaceae* | *Bacteroides* | *B.thetaiotaomicron* | Decrease |  |
|  | *Bifidobacteriaceae* | *Bifidobacterium* |  | Decrease |  |
|  | *Enterococcaceae* | *Enterococcus* |  | Decrease |  |
|  | *Lachnospiraceae* | *Blautia* |  | Decrease |  |
|  |  | *Butyrivibrio* |  | Decrease |  |
|  |  | *Coprococcus* |  | Decrease |  |
|  |  | *Roseburia* | *R.hominis* | Decrease |  |
|  |  |  | *R.intestinalis,* | Decrease |  |
|  | *Oscillospiraceae* | *Faecalibacterium* | *F.prausnitzii* | Decrease |  |
|  |  | *Oscillibacter* |  | *Decrease* |  |
|  | *Synergistaceae* | *Synergistetes* |  | Decrease |  |
| Heart failure | *Acidaminococcaceae* | *Succiclasticum* |  | Increase | (6-10) |
|  | *Campylobacteraceae* | *Campylobacter* |  | Increase |  |
|  | *Clostridiaceae* | *Hungatella* |  | Increase |  |
|  | *Debaryomycetaceae* | *Candida* |  | Increase |  |
|  | *Enterobacteriaceae* | *Salmonella* |  | Increase |  |
|  | *Enterobacteriaceae* | *Shigella* |  | Increase |  |
|  | *Ruminococcaceae* | *Ruminococcus* | *R.gnavus* | Increase |  |
|  | *Prevotellaceae* | *Prevotella* |  | Increase |  |
|  | *Yersiniaceae* | *Yersinia* | *Y.enterocolitica* | Increase |  |
|  | *Bifidobacteriaceae* | *Bifidobacterium* |  | Decrease |  |
|  | *Coriobacteriaceae,* | *Collinsella* |  | Decrease |  |
|  | *Erysipelotrichaceae* |  |  | Decrease |  |
|  | *Lachnospiraceae* | *Anaerostipes* |  | Decrease |  |
|  |  | *Blautia* |  | Decrease |  |
|  |  | *Coprococcus* |  | Decrease |  |
|  | *Oscillospiraceae* | *Faecalibacterium* | *F.prausnitzii* | Decrease |  |
|  | *Ruminococcaceae* |  |  | Decrease |  |
| Myocardial infarction | *Eggerthellaceae* | *Eggerthella* | *E.lenta* | Increase | (11,12) |
|  | *Enterobacteriaceae* | *Klebsiella* | *K.Pneumoniae* | Increase |  |
|  |  | *Escherichia* | *E.coli* | Increase |  |
|  |  | *Enterobacter* | *E.aerogenes* | Increase |  |
|  | *Streptococcaceae* | *Streptococcus* | *S.salivarius* | Increase |  |
|  | *Lachnospiraceae* | *Roseburia* | *R.intestinalis* | Decrease |  |
|  | *Oscillospiraceae* | *Faecalibacterium* | *F.cf.prausnitzii* | Decrease |  |
|  | *Bacteroidaceae* | *Fusobacterium* |  | Decrease |  |
| Atrial fibrillation | *Clostridiaceae* | *Collagenase* | *C.coprobacillus* | Increase | (13,14) |
|  | *Enterococcaceae* | *Enterococcus* |  | Increase |  |
|  | *Ruminococcaceae* | *Ruminococcus* |  | Increase |  |
|  | *Streptococcaceae* | *Streptococcus* |  | Increase |  |
|  | *Tannerellaceae* | *Parabacteroides* |  | Increase |  |
|  | *Veillonellaceae* | *Veillonella* |  | Increase |  |
|  | *Desulfovibrionaceae* | *Bilophila* |  | Decrease |  |
|  | *Oscillospiraceae* | *Faecalibacterium* | *F.prausnitzii* | Decrease |  |
|  |  | *Oscillibacter* |  | Decrease |  |
|  | *Rikenellaceae* | *Alistipes* |  | Decrease |  |
| Chronic kidney disease | *Clostridiaceae* | *Clostridium* | *C.perfringens* | Increase | (15,16) |
|  | *Enterobacteriaceae* | *Enterobacteria* |  | Increase |  |
|  | *Enterococcaceae* | *Enterococci* |  | Increase |  |
|  | *Erysipelotrichidae* | *Catenibacterium* |  | Increase |  |
|  | *Halomonadaceae* |  |  | Increase |  |
|  | *Micrococcaceae* | *Nesterenkonia* |  | Increase |  |
|  | *Moraxellaceae* |  |  | Increase |  |
|  | *Polyangiaceae* |  |  | Increase |  |
|  | *Pseudomonadaceae* |  |  | Increase |  |
|  | *Thiothrix* |  |  | Increase |  |
|  | *Bifidobacteriaceae* | *Bifidobacterium* |  | Decrease |  |

**Supplementary Table 2.** Bacterial strains with either the *CutC*/*D* or *CntA*/*B* and their growing conditions

| Genes | Organism | Growing conditions | References |
| --- | --- | --- | --- |
| *CutC*/*D* | *Desulfovibrio alaskensis* | Obligate anaerobic | (17) |
|  | *Escherichia coli* | Facultative anaerobic |  |
|  | *Olesnella uli* | Aerobic |  |
|  | *Pelobacter acetylenicus* | Obligate anaerobic |  |
|  | *Pelobacter carbinolicus* | Obligate anaerobic |  |
|  | *Proteus mirabilis* | Facultative anaerobic |  |
|  | *Proteus penneri* | Facultative anaerobic |  |
|  | *Proteus vulgaris* | Facultative anaerobic |  |
|  | *Vibrio cholinicus* | Aerobic |  |
|  | *Alkaliphilus spp.* | Obligate anaerobic | (18) |
|  | *Bacteroides spp.* | Obligate anaerobic |  |
|  | *Blautia spp.* | Obligate anaerobic |  |
|  | *Cetobacterium spp.* | Aerobic |  |
|  | *Collinsella spp.* | Anaerobic |  |
|  | *Enterococcus spp.* | Facultative anaerobic |  |
|  | *Flavonifractor spp.* | Obligate anaerobic |  |
|  | *Gemmiger spp.* | Anaerobic |  |
|  | *Johnsonella spp.* | Anaerobic |  |
|  | *Romboutsia spp.* | Anaerobic |  |
|  | *Clostridium sporogenes* | Obligate anaerobic | (19) |
|  | *Clostridium tetani* | Obligate anaerobic |  |
|  | *Klebsiella oxytoca* | Facultative anaerobic |  |
|  | *Klebsiella pneumoniae* | Facultative anaerobic |  |
|  | *Klebsiella variicola* | Facultative anaerobic |  |
|  | *Providencia rettgeri* | Anaerobic |  |
|  | *Desulfovibrio desulfuricans* | Obligate anaerobic | (20) |
|  | *Anaerococcus hydrogenalis* | Anaerobic | (21) |
|  | *Clostridium asparagiforme* | Obligate anaerobic |  |
|  | *Clostridium hathewayi* | Obligate anaerobic |  |
|  | *Edwardsiella tarda* | Facultative anaerobic |  |
|  | *Escherichia fergusonii* | Aerobic |  |
| *CntA*/*B* | *Acinetobacter calcoaceticus* | Aerobic | (17) |
|  | *Citrobacter freundii* | Facultative anaerobic |  |
|  | *Escherichia coli* | Facultative anaerobic |  |
|  | *Providencia rettgeri* | Anaerobic |  |
|  | *Serratia marcescens* | Facultative anaerobic |  |
|  | *Burkholderia spp.* | Aerobic | (18) |
|  | *Cupriavidus spp.* | Aerobic |  |
|  | *Pseudomonas spp.* | Obligate aerobic |  |
|  | *Stenotrophomonas spp.* | Aerobic |  |
|  | *Yersinia spp.* | Facultative anaerobic |  |
|  | *Yokenella spp.* | Aerobic |  |
|  | *Acinetobacter baumannii* | Obligate aerobic | (22) |
|  | *Klebsiella pneumoniae* | Facultative anaerobic | (23) |
|  | *Shigella spp.* | Facultative anaerobic |  |
|  | *Sporosarcina spp.* | Aerobiotic |  |

**Supplementary Table 3.** The specific mechanisms by which TMAO is involved in cardiovascular diseases

| Cardiovascular diseases | Mechanism of TMAO promoting cardiovascular diseases | References |
| --- | --- | --- |
| Atherosclerosis | TMAO was negatively correlated with levels of high-density lipoprotein cholesterol of patients with atherosclerotic cardiovascular disease; High-density lipoprotein was lower and plasma TMAO levels were significantly higher in non‑alcoholic fatty liver disease patients. | (24,25) |
|  | TMAO suppressed reverse cholesterol transport and affected cholesterol metabolism. | (26) |
|  | CD36 expression was enhanced in ApoE^-/-^ mice given water with TMAO; TMAO increased the levels of scavenger receptor cluster of differentiation 36 (CD36) and scavenger receptor-A1 (SR-A1) in the macrophages of mice. | (27,28) |
|  | The expression of ATP-binding cassette transporter A1 (ABCA1) was downregulated in murine macrophage J774A.1 cells treated with TMAO; Dietary supplementation with TMAO significantly decreased the expression of ABCG5/G8, which lowered the transport of cholesterol to gut lumen in germ-free mice. | (29,30) |
|  | TMAO directly triggered intracellular Ca^2+^ release from internal stores, increasing platelet hyperactivity and enhancing thrombosis potential. | (31-33) |
|  | TMAO decreased the gene expression of hepatic BAs synthase cytochrome P450 family 7 subfamily A member 1 (CYP7A1) and cytochrome P450 family 27 subfamily A member 1 (CYP27A1) in ApoE^-/-^ mice. | (34) |
|  | Choline diet elevated the risks of foam cell formation in murine macrophages; TMAO facilitated the formation of foam cell in human monocyte cell lines. | (35,36) |
|  | Increased TMAO triggered nuclear factor-kappa B (NF-κB) in aortic valve interstitial cells (AVICs). | (37,38) |
|  | TMAO increased the expression of pro-inflammatory cytokines, such as TNF-α and IL-1B, and decreased anti-inflammatory cytokines such as IL-10. | (39,40) |
|  | TMAO elevated ROS levels and downregulated the expression of SIRT1 in vascular smooth muscle cells (VSMCs) and human umbilical vein endothelial cells (HUVECs). | (41) |
|  | The oxidative stress was triggered by TMAO, which inhibited the production of nitric oxide (NO) and endothelial nitric oxide synthase (eNOS); TMAO activated TXNIP-NLRP3 inflammasome and increased the expression of IL-1β and IL-18. | (42,43) |
|  | TMAO stimulated the high mobility group box 1 (HMGB1) production and inhibited the expression of junction proteins like zonula occludens-2 (ZO-2), occludin, and vascular endothelial cadherin (VE-cadherin) in endothelial cells compared with control cells. | (44,45) |
|  | The expression of vascular cell adhesion molecule-1 (VCAM-1) were increased by TMAO, leading to endothelial cell dysfunction and promoting the adhesion of monocytes. | (46) |
| Heart failure | TMAO activated the small mothers against decapentaplegic 3 (Smad3) signaling pathway and increased the expression of atrial natriuretic peptide (ANP) and beta-myosin heavy chain (β-MHC), stimulating cardiac hypertrophy and perivascular fibrosis in SD rats. | (47) |
|  | Mice fed on diets supplemented with high sugar and fat had elevated plasma TMAO levels and increased TNF-α levels. | (48) |
|  | TMAO damaged cardiac compliance and function. | (34) |
|  | TMAO promoted mitochondrial dysfunction, decreasing contractility of cardiomyocytes. | (49,50) |
|  | The activity of pyruvate dehydrogenase (PDH) was inhibited by TMAO, which decreased production of ATP. | (51) |
|  | TMAO led to the accumulation of glycogen and the deposition of lipofuscin-like pigment in the paranuclear area of cardiomyocytes, damaging the function of mitochondria, proteasome system and lysosomal. | (52) |
|  | Increase of TMAO induced transverse-tubule (T-tubule) damage, promoted Junctophilin2 (JPH2) redistribution and led to Ca^2+^ handling dysfunction in cardiomyocytes; High-sugar-and-high-fat-induced TMAO led to oxidative dysfunction and Ca^2+^-disorder. | (53,54) |
|  | TMAO caused the renal interstitial fibrosis and dysfunction, promoting sodium and water retention, which indirectly worsened heart failure. | (55,56) |
| Hypertension | People with high concentrations of TMAO had a 12% increased risk of hypertension compared with those with low concentrations of TMAO, and the risk of hypertension increased by 20% for every 10μmol/L increase in TMAO. | (57) |
|  | Structure of Angiotensin II (Ang II) was influenced by TMAO and increased cardiovascular risks; TMAO acted on Angiotensin II, promoted vasoconstriction and induced hypertension in mice. | (58,59) |
|  | TMAO increased plasma osmotic pressure and triggered the regulation of the TMAO-AVP-AQP-2 axis, increasing water reabsorption in spontaneously hypertensive rats. | (60) |
|  | TMAO increased hypertension risks by triggering inflammatory responses and endothelial dysfunction. | (61) |

**Supplementary Table 4.** Ways to reduce the levels of TMA/TMAO

| Object | Test subjects | Treatment | Results | References |
| --- | --- | --- | --- | --- |
| L-carnitine TMA lyase | Wistar rats | Administration with L-carnitine, choline alone or in combination with 0.5% meldonium | Treatment with meldonium significantly decreased production of TMA from L-carnitine by intestinal microbiota | (62) |
| Choline TMA lyase | Mice | Co-feeding mice with 1% choline supplement and 1% (v/v) DMB in the drinking water | Reduced circulating levels of TMAO in mice and attenuated foam cells formation in ApoE^-/-^ mice fed a high choline diet | (63) |
| TMA/TMAO-degrading strains | Mice | Oral administration of TMA metabolizing strain *Enterobacter aerogenes* ZDY01 | Reduced levels of TMA in cecum and TMAO in serum | (64) |
| FMO3 | Mice of insulin-intolerant | The use of 3,3′-diindolylmethane or indole-3-carbinol as a FMO3 inhibitor | Reduced TMAO levels and insulin resistance | (65) |
| Gut microbiota | Rats with myocardial ischemia | Supplement of *Lactobacillus rhamnosus* GR-1 in the drinking water of rats | Significantly improved left ventricular ejection fraction of rats | (66) |
|  | People with heart failure | A 3-month daily therapy with *Saccharomyces Boulardii* to volunteers | Reduced systemic inflammation and improved left ventricular ejection fraction | (67) |
|  | Healthy people | Supplement of *Bifidobacterium animalis subsp. lactis* LKM512 to volunteers | Reduced fecal TMA concentrations | (68) |
|  | People who were high-TMAO  producers | An intervention of raw garlic juice for a week to volunteers | Improved gut microbiota variety and increased relative abundance of beneficial bacteria | (69) |
|  | ApoE^-/-^ mice | feeding mice with resveratrol | Reduced TMAO levels by decreasing TMA generation via remodeling microbiota, and increased hepatic BA synthesis | (70) |
| Fecal microbiota transplantation | People | A fecal microbiota transplantation strategy for obese patients with metabolic syndrome | A temporary normalized insulin sensitivity in obese subjects and improved levels of  butyrate-producing intestinal microbiota | (71) |
| Diet | People | An adoption of Mediterranean diet with extra-virgin olive oil, nuts, or reduced-fat diet to participants. | Supplement with extra-virgin olive oil or nuts lowered incidence of major cardiovascular events than a reduced-fat diet | (72) |

**Reference**

1. Jie Z, Xia H, Zhong SL, Feng Q, Li S, Liang S, et al. The Gut Microbiome in Atherosclerotic Cardiovascular Disease. Nat Commun (2017) 8:845. doi: 10.1038/s41467-017-00900-1

2. Duttaroy AK. Role of Gut Microbiota and Their Metabolites on Atherosclerosis, Hypertension and Human Blood Platelet Function: A Review. Nutrients (2021) 13:144. doi: 10.3390/nu13010144

3. Li J, Zhao F, Wang Y, Chen J, Tao J, Tian G, et al. Gut Microbiota Dysbiosis Contributes to the Development of Hypertension. Microbiome (2017) 5:14. doi: 10.1186/s40168-016-0222-x

4. Yan Q, Gu Y, Li X, Yang W, Jia L, Chen C, et al. Alterations of the Gut Microbiome in Hypertension. Front Cell Infect Microbiol (2017) 7:381. doi: 10.3389/fcimb.2017.00381

5. Naqvi S, Asar TO, Kumar V, Al-Abbasi FA, Alhayyani S, Kamal MA, et al. A Cross-Talk Between Gut Microbiome, Salt and Hypertension. Biomed Pharmacother (2021) 134:111156. doi: 10.1016/j.biopha.2020.111156

6. Pasini E, Aquilani R, Testa C, Baiardi P, Angioletti S, Boschi F, et al. Pathogenic Gut Flora in Patients with Chronic Heart Failure. JACC Heart Fail (2016) 4:220-7. doi: 10.1016/j.jchf.2015.10.009

7. Tang WHW, Li DY, Hazen SL. Dietary Metabolism, the Gut Microbiome, and Heart Failure. Nat Rev Cardiol (2019) 16:137-54. doi: 10.1038/s41569-018-0108-7

8. Luedde M, Winkler T, Heinsen FA, Rühlemann MC, Spehlmann ME, Bajrovic A, et al. Heart Failure is Associated with Depletion of Core Intestinal Microbiota. ESC Heart Fail (2017) 4:282-90. doi: 10.1002/ehf2.12155

9. Mayerhofer CCK, Kummen M, Holm K, Broch K, Awoyemi A, Vestad B, et al. Low Fibre Intake is Associated with Gut Microbiota Alterations in Chronic Heart Failure. ESC Heart Fail (2020) 7:456-66. doi: 10.1002/ehf2.12596

10. Chaikijurajai T, Tang WHW. Gut Microbiome and Precision Nutrition in Heart Failure: Hype or Hope? Curr Heart Fail Rep (2021) 18:23-32. doi: 10.1007/s11897-021-00503-4

11. Han Y, Gong Z, Sun G, Xu J, Qi C, Sun W, et al. Dysbiosis of Gut Microbiota in Patients with Acute Myocardial Infarction. Front Microbiol (2021) 12:680101. doi: 10.3389/fmicb.2021.680101

12. Vahed SZ, Barzegari A, Zuluaga M, Letourneur D, Pavon-Djavid G. Myocardial Infarction and Gut Microbiota: An Incidental Connection. Pharmacol Res (2018) 129:308-17. doi: 10.1016/j.phrs.2017.11.008

13. Zuo K, Li J, Li K, Hu C, Gao Y, Chen M, et al. Disordered Gut Microbiota and Alterations in Metabolic Patterns are Associated with Atrial Fibrillation. Gigascience (2019) 8:giz058. doi: 10.1093/gigascience/giz058

14. Tabata T, Yamashita T, Hosomi K, Park J, Hayashi T, Yoshida N, et al. Gut Microbial Composition in Patients with Atrial Fibrillation: Effects of Diet and Drugs. Heart Vessels (2021) 36:105-14. doi: 10.1007/s00380-020-01669-y

15. Vaziri ND, Wong J, Pahl M, Piceno YM, Yuan J, DeSantis TZ, et al. Chronic Kidney Disease Alters Intestinal Microbial Flora. Kidney Int (2013) 83:308-15. doi: 10.1038/ki.2012.345

16. Rysz J, Franczyk B, Ławiński J, Olszewski R, Ciałkowska-Rysz A, Gluba-Brzózka A. The Impact of CKD on Uremic Toxins and Gut Microbiota. Toxins（basel） (2021) 13:252. doi: 10.3390/toxins13040252

17. Jameson E, Quareshy M, Chen Y. Methodological considerations for the identification of choline and carnitine-degrading bacteria in the gut. *Methods*. (2018) 149:42-8. doi: 10.1016/j.ymeth.2018.03.012

18. Cai YY, Huang FQ, Lao X, Lu Y, Gao X, Alolga RN, et al. Author Correction: Integrated metagenomics identifies a crucial role for trimethylamine-producing Lachnoclostridium in promoting atherosclerosis. *NPJ Biofilms Microbiomes*. (2022) 8:40. doi: 10.1038/s41522-022-00303-1

19. Ramireddy L, Tsen HY, Chiang YC, Hung CY, Chen FC, Yen HT. The gene expression and bioinformatic analysis of choline trimethylamine-lyase (CutC) and its activating enzyme (CutD) for gut microbes and comparison with their TMA production levels. *Curr Res Microb Sci*. (2021) 2:100043. doi: 10.1016/j.crmicr.2021.100043

20. Craciun S, Balskus EP. Microbial conversion of choline to trimethylamine requires a glycyl radical enzyme. *Proc Natl Acad Sci U S A*. (2012) 109:21307-12. doi: 10.1073/pnas.1215689109

21. Romano KA, Vivas EI, Amador-Noguez D, Rey FE. Intestinal microbiota composition modulates choline bioavailability from diet and accumulation of the proatherogenic metabolite trimethylamine-N-Oxide. *Mbio*. (2015) 6:e2481. doi: 10.1128/mBio.02481-14

22. Massmig M, Reijerse E, Krausze J, Laurich C, Lubitz W, Jahn D, et al. Carnitine metabolism in the human gut: Characterization of the two-component carnitine monooxygenase CntAB from Acinetobacter baumannii. *J Biol Chem*. (2020) 295:13065-78. doi: 10.1074/jbc.RA120.014266

23. Zeisel SH, Warrier M. Trimethylamine N-Oxide, the microbiome, and heart and kidney disease. *Annu Rev Nutr*. (2017) 37:157-81. doi: 10.1146/annurev-nutr-071816-064732

24. Xiong X, Zhou J, Fu Q, Xu X, Wei S, Yang S, et al. The Associations Between TMAO-Related Metabolites and Blood Lipids and the Potential Impact of Rosuvastatin Therapy. Lipids Health Dis (2022) 21:60. doi: 10.1186/s12944-022-01673-3

25. Moradzad M, Abdi M, Esmaeili FS, Ghaderi D, Rahmani K, Moloudi MR, et al. Possible Correlation Between High Circulatory Levels of Trimethylamine-N-Oxide and 2177G>C Polymorphisms of Hepatic Flavin Containing Monooxygenase 3 in Kurdish Population with Non-Alcoholic Fatty Liver Disease. Mol Biol Rep (2022) 49:5927-37. doi: 10.1007/s11033-022-07375-4

26. Koeth RA, Levison BS, Culley MK, Buffa JA, Wang Z, Gregory JC, et al. γ- Butyrobetaine is a Proatherogenic Intermediate in Gut Microbial Metabolism of L-carnitine to TMAO. Cell Metab (2014) 20:799-812. doi: 10.1016/j.cmet.2014.10.006

27. Wang Z, Klipfell E, Bennett BJ, Koeth R, Levison BS, Dugar B, et al. Gut Flora Metabolism of Phosphatidylcholine Promotes Cardiovascular Disease. Nature (2011) 472:57-63. doi: 10.1038/nature09922

28. Geng J, Yang C, Wang B, Zhang X, Hu T, Gu Y, et al. Trimethylamine N-Oxide Promotes Atherosclerosis C *via* D36-Dependent MAPK/JNK Pathway. Biomed Pharmacother (2018) 97:941-7. doi: 10.1016/j.biopha.2017.11.016

29. Mohammadi A, Najar AG, Yaghoobi MM, Jahani Y, Vahabzadeh Z. Trimethylamine-N-Oxide Treatment Induces Changes in the ATP-Binding Cassette Transporter A1 and Scavenger Receptor A1 in Murine Macrophage J774A.1 Cells. Inflammation (2016) 39:393-404. doi: 10.1007/s10753-015-0261-7

30. Koeth RA, Wang Z, Levison BS, Buffa JA, Org E, Sheehy BT, et al. Intestinal Microbiota Metabolism of L-Carnitine, a Nutrient in Red Meat, Promotes Atherosclerosis. Nat Med (2013) 19:576-85. doi: 10.1038/nm.3145

31. Ding L, Chang M, Guo Y, Zhang L, Xue C, Yanagita T, et al. Trimethylamine-N-Oxide (TMAO)-Induced Atherosclerosis is Associated with Bile Acid Metabolism. Lipids Health Dis (2018) 17:286. doi: 10.1186/s12944-018-0939-6

32. Zhu W, Gregory JC, Org E, Buffa JA, Gupta N, Wang Z, et al. Gut Microbial Metabolite TMAO Enhances Platelet Hyperreactivity and Thrombosis Risk. Cell (2016) 165:111-24. doi: 10.1016/j.cell.2016.02.011

33. Roberts AB, Gu X, Buffa JA, Hurd AG, Wang Z, Zhu W, et al. Development of a Gut Microbe-Targeted Nonlethal Therapeutic to Inhibit Thrombosis Potential. Nat Med (2018) 24:1407-17. doi: 10.1038/s41591-018-0128-1

34. Guasch-Ferré M, Hu FB, Ruiz-Canela M, Bulló M, Toledo E, et al. Plasma Metabolites from Choline Pathway and Risk of Cardiovascular Disease in the PREDIMED (Prevention with Mediterranean Diet) Study. J Am Heart Assoc (2017) 6:e006524. doi: 10.1161/JAHA.117.006524

35. Ahmadi A, Vahabzadeh Z, Moloudi M, Farhadi L, Shirahmadi S. Contribution of Toll-Like Receptor 2 and Nicotinamide Adenine Dinucleotide Phosphate Oxidase to the Trimethylamine N-Oxide-Induced Inflammatory Reactions in U937-Derived Macrophages. ARYA Atheroscler (2021) 17:1-7. doi: 10.22122/arya.v17i0.2096

36. Velasquez MT, Ramezani A, Manal A, Raj DS. Trimethylamine N-Oxide: The Good, the Bad and the Unknown. Toxins (Basel) (2016) 8:326. doi: 10.3390/toxins8110326

37. Li J, Zeng Q, Xiong Z, Xian G, Liu Z, Zhan Q, et al. Trimethylamine N-Oxide Induces Osteogenic Responses in Human Aortic Valve Interstitial Cells in *Vitro* and Aggravates Aortic Valve Lesions in Mice. Cardiovasc Res (2022) 118:2018-30. doi: 10.1093/cvr/cvab243

38. Zhang X, Li Y, Yang P, Liu X, Lu L, Chen Y, et al. Trimethylamine-N-Oxide Promotes Vascular Calcification Through Activation of NLRP3 (Nucleotide-Binding Domain, Leucine-Rich-Containing Family, Pyrin Domain-Containing-3) Inflammasome and NF-KappaB (Nuclear Factor KappaB) Signals. Arterioscler Thromb Vasc Biol (2020) 40:751-65. doi: 10.1161/ATVBAHA.119.313414

39. Chen K, Zheng X, Feng M, Li D, Zhang H. Gut Microbiota-Dependent Metabolite Trimethylamine N-Oxide Contributes to Cardiac Dysfunction in Western Diet-Induced Obese Mice. Front Physiol (2017) 8:139. doi: 10.3389/fphys.2017.00139

40. Zhang L, Xie F, Tang H, Zhang X, Hu J, Zhong X, et al. Gut Microbial Metabolite TMAO Increases Peritoneal Inflammation and Peritonitis Risk in Peritoneal Dialysis Patients. Transl Res (2022) 240:50-63. doi: 10.1016/j.trsl.2021.10.001

41. Zhou S, Xue J, Shan J, Hong Y, Zhu W, Nie Z, et al. Gut-Flora-Dependent Metabolite Trimethylamine-N-Oxide Promotes Atherosclerosis-Associated Inflammation Responses by Indirect ROS Stimulation and Signaling Involving AMPK and SIRT1. Nutrients (2022) 14:3338. doi: 10.3390/nu14163338

42. Sun X, Jiao X, Ma Y, Liu Y, Zhang L, He Y, et al. Trimethylamine N-Oxide Induces Inflammation and Endothelial Dysfunction in Human Umbilical Vein Endothelial Cells *via* Activating ROS-TXNIP-NLRP3 Inflammasome. Biochem Biophy Res Commun (2016) 481:63-70. doi: 10.1016/j.bbrc.2016.11.017

43. Querio G, Antoniotti S, Geddo F, Levi R, Gallo MP. Trimethylamine N-Oxide (TMAO) Impairs Purinergic Induced Intracellular Calcium Increase and Nitric Oxide Release in Endothelial Cells. Int J Mol Sci (2022) 23:3982. doi: 10.3390/ijms23073982

44. Singh GB, Zhang Y, Boini KM, Koka S. High Mobility Group Box 1 Mediates TMAO-Induced Endothelial Dysfunction. Int J Mol Sci (2019) 20:3570. doi: 10.3390/ijms20143570

45. Liu Y, Dou C, Wei G, Zhang L, Xiong W, Wen L, et al. *Usnea* Improves High-Fat Diet- and Vitamin D3-Induced Atherosclerosis in Rats by Remodeling Intestinal Flora Homeostasis. Front Pharmacol (2022) 13:1064872. doi: 10.3389/fphar.2022.1064872

46. Ma G, Pan B, Chen Y, Guo C, Zhao M, Zheng L, et al. Trimethylamine N-Oxide in Atherogenesis: Impairing Endothelial Self-Repair Capacity and Enhancing Monocyte Adhesion. Biosci Rep (2017) 37:BSR20160244. doi: 10.1042/BSR20160244

47. Li Z, Wu Z, Yan J, Liu H, Liu Q, Deng Y, et al. Gut Microbe-Derived Metabolite Trimethylamine N-Oxide Induces Cardiac Hypertrophy and Fibrosis. Lab Invest (2019) 99:346-57. doi: 10.1038/s41374-018-0091-y

48. Zhang H, Meng J, Yu H. Trimethylamine N-Oxide Supplementation Abolishes the Cardioprotective Effects of Voluntary Exercise in Mice Fed a Western Diet. Front Physiol (2017) 8:944. doi: 10.3389/fphys.2017.00944

49. Savi M, Bocchi L, Bresciani L, Falco A, Quaini F, Mena P, et al. Trimethylamine-N-Oxide (TMAO)-Induced Impairment of Cardiomyocyte Function and the Protective Role of Urolithin B-Glucuronide. Molecules (2018) 23:549. doi: 10.3390/molecules23030549

50. Zabell A, Tang WHW. Targeting the Microbiome in Heart Failure. Curr Treat Options Cardiovasc Med (2017) 19:27. doi: 10.1007/s11936-017-0528-4

51. Makrecka-Kuka M, Volska K, Antone U, Vilskersts R, Grinberga S, Bandere D, et al. Trimethylamine N-Oxide Impairs Pyruvate and Fatty Acid Oxidation in Cardiac Mitochondria. Toxicol Lett (2017) 267:32-8. doi: 10.1016/j.toxlet.2016.12.017

52. Jung T, Bader N, Grune T. Lipofuscin: Formation, Distribution, and Metabolic Consequences. Ann N Y Acad Sci (2007) 1119:97-111. doi: 10.1196/annals.1404.008

53. Jin B, Ji F, Zuo A, Liu H, Qi L, He Y, et al. Destructive Role of TMAO in T-Tubule and Excitation-Contraction Coupling in the Adult Cardiomyocytes. Int Heart J (2020) 61:355-63. doi: 10.1536/ihj.19-372

54. Singh RB, Fedacko J, Pella D, Fatima G, Elkilany G, Moshiri M, et al. High Exogenous Antioxidant, Restorative Treatment (Heart) for Prevention of the Six Stages of Heart Failure: The Heart Diet. Antioxidants (Basel) (2022) 11:1464. doi: 10.3390/antiox11081464

55. Li XS, Obeid S, Klingenberg R, Gencer B, Mach F, Räber L, et al. Gut Microbiota-Dependent Trimethylamine N-Oxide in Acute Coronary Syndromes: A Prognostic Marker for Incident Cardiovascular Events Beyond Traditional Risk Factors. Eur Heart J (2017) 38:814-24. doi: 10.1093/eurheartj/ehw582

56. Zou D, Li Y, Sun G. Attenuation of Circulating Trimethylamine N-Oxide Prevents the Progression of Cardiac and Renal Dysfunction in a Rat Model of Chronic Cardiorenal Syndrome. Front Pharmacol (2021) 12:751380. doi: 10.3389/fphar.2021.751380

57. Ge X, Zheng L, Zhuang R, Yu P, Xu Z, Liu G, et al. The Gut Microbial Metabolite Trimethylamine N-Oxide and Hypertension Risk: A Systematic Review and Dose-Response Meta-Analysis. Adv Nutr (2020) 11:66-76. doi: 10.1093/advances/nmz064

58. Jiang S, Shui Y, Cui Y, Tang C, Wang X, Qiu X, et al. Gut Microbiota Dependent Trimethylamine N-Oxide Aggravates Angiotensin II–Induced Hypertension. Redox Biol (2021) 46:102115. doi: 10.1016/j.redox.2021.102115

59. Ufnal M, Jazwiec R, Dadlez M, Drapala A, Sikora M, Skrzypecki J. Trimethylamine-N-Oxide: A Carnitine-Derived Metabolite that Prolongs the Hypertensive Effect of Angiotensin II in Rats. Can J Cardiol (2014) 30:1700-5. doi: 10.1016/j.cjca.2014.09.010

60. Liu M, Han Q, Yang J. Trimethylamine-N-Oxide (TMAO) Increased Aquaporin-2 Expression in Spontaneously Hypertensive Rats. Clin Exp Hypertens (2019) 41:312-22. doi: 10.1080/10641963.2018.1481420

61. Zhou W, Cheng Y, Zhu P, Nasser MI, Zhang X, Zhao M. Implication of Gut Microbiota in Cardiovascular Diseases. Oxid Med Cell Longev (2020) 2020:5394096. doi: 10.1155/2020/5394096

62. Kuka J, Liepinsh E, Makrecka-Kuka M, Liepins J, Cirule H, Gustina D, et al. Suppression of Intestinal Microbiota-Dependent Production of Pro-Atherogenic Trimethylamine N-Oxide by Shifting L-Carnitine Microbial Degradation. Life Sci (2014) 117:84-92. doi: 10.1016/j.lfs.2014.09.028

63. Wang Z, Roberts AB, Buffa JA, Levison BS, Zhu W, Org E, et al. Non-Lethal Inhibition of Gut Microbial Trimethylamine Production for the Treatment of Atherosclerosis. Cell (2015) 163:1585-95. doi: 10.1016/j.cell.2015.11.055

64. Qiu L, Yang D, Tao X, Yu J, Xiong H, Wei H. *Enterobacter Aerogenes* ZDY01 Attenuates Choline-Induced Trimethylamine N-Oxide Levels by Remodeling Gut Microbiota in Mice. J Microbiol Biotechnol (2017) 27:1491-9. doi: 10.4014/jmb.1703.03039

65. Simó C, García-Cañas V. Dietary Bioactive Ingredients to Modulate the Gut Microbiota-Derived Metabolite TMAO. New Opportunities for Functional Food Development. Food Funct (2020) 11:6745-76. doi: 10.1039/d0fo01237h

66. Gan XT, Ettinger G, Huang CX, Burton JP, Haist JV, Rajapurohitam V, et al. Probiotic Administration Attenuates Myocardial Hypertrophy and Heart Failure After Myocardial Infarction in the Rat. Circ Heart Fail (2014) 7:491-9. doi: 10.1161/CIRCHEARTFAILURE.113.000978

67. Costanza AC, Moscavitch SD, Faria Neto HCC, Mesquita ET. Probiotic Therapy with *Saccharomyces Boulardii* for Heart Failure Patients: A Randomized, Double-Blind, Placebo-Controlled Pilot Trial. Int J Cardiol (2015) 179:348-50. doi: 10.1016/j.ijcard.2014.11.034

68. Matsumotoa M, Kitada Y, Shimomura Y, Naito Y. *Bifidobacterium Animalis* *Subsp. Lactis* LKM512 Reduces Levels of Intestinal Trimethylamine Produced by Intestinal Microbiota in Healthy Volunteers: A Double-Blind, Placebo-Controlled Study. J Func Foods (2018) 36:94-101. doi: 10.1016/j.jff.2017.06.032

69. Panyod S, Wu WK, Chen PC, Chong KV, Yang YT, Chuang HL, et al. Atherosclerosis Amelioration by Allicin in Raw Garlic Through Gut Microbiota and Trimethylamine-N-Oxide Modulation. NPJ Biofilms Microbiomes (2022) 8:4. doi: 10.1038/s41522-022-00266-3

70. Chen ML, Yi L, Zhang Y, Zhou X, Ran L, Yang J, et al. Resveratrol Attenuates Trimethylamine-N-Oxide (TMAO)-Induced Atherosclerosis by Regulating TMAO Synthesis and Bile Acid Metabolism *via* Remodeling of the Gut Microbiota. MBio (2016) 7:e02210-15. doi: 10.1128/mBio.02210-15

71. Vrieze A, Van Nood E, Holleman F, Salojärvi J, Kootte RS, Bartelsman JFWM, et al. Transfer of Intestinal Microbiota from Lean Donors Increases Insulin Sensitivity in Individuals with Metabolic Syndrome. Gastroenterology (2012) 143:913-6. doi: 10.1053/j.gastro.2012.06.031

72. Estruch R, Ros E, Salas-Salvadó J, Covas MI, Corella D, Arós F, et al. Primary Prevention of Cardiovascular Disease with a Mediterranean Diet Supplemented with Extra-Virgin Olive Oil or Nuts. N Engl J Med (2018) 378:e34. doi: 10.1056/NEJMoa1800389
